# Estimation of mean erythrocyte age using HbA1c or HbA1c/glycated albumin for evaluation of anemia severity

**DOI:** 10.1002/jcla.24947

**Published:** 2023-07-30

**Authors:** Masafumi Koga, Masahi Kameyama, Toshika Okumiya

**Affiliations:** ^1^ Department of Internal Medicine Hakuhokai Central Hospital Amagasaki Japan; ^2^ Research Team for Neuroimaging Tokyo Metropolitan Institute for Geriatrics and Gerontology Tokyo Japan; ^3^ Department of Medical Laboratory Science, Faculty of Health Sciences Kochi Gakuen University Kochi Japan

**Keywords:** erythrocyte creatine, glycated albumin, HbA1c, hemolytic anemia, mean erythrocyte age

## Abstract

**Background:**

Hemoglobin A1c (HbA1c) levels are low in patients with hemolytic anemia, as HbA1c reflects mean erythrocyte age (M_RBC_). Erythrocyte creatine (EC) is a hemolytic indicator that also reflects M_RBC_. We previously reported an equation for estimating M_RBC_ using EC (EC‐M_RBC_).

**Aims:**

In this study, EC‐M_RBC_ was compared to the HbA1c level expressed in the International Federation of Clinical Chemistry and Laboratory Medicine units (iA1c) and to the iA1c/glycated albumin (GA) ratio to estimate M_RBC_.

**Methods:**

This study included 238 subjects, including patients with hemolytic anemia and/or type 2 diabetes mellitus (T2DM).

**Results:**

In non‐diabetic individuals, both iA1c and iA1c/GA showed a strong positive correlation with EC‐M_RBC_ (*p* < 0.0001). The equations to estimate iA1c‐M_RBC_ and iA1c/GA‐M_RBC_ derived from the regression equations between EC‐M_RBC_ and iA1c, and EC‐M_RBC_ and iA1c/GA in nondiabetic individuals were 1.45 × iA1c and 20.0 × iA1c/GA, respectively. iA1c‐MRBC and iA1c/GA‐MRBC in non‐diabetic individuals without hemolytic anemia were 57.6 ± 4.0 and 57.1 ± 6.4 days, respectively, and iA1c/GA‐MRBC in T2DM patients without hemolytic anemia was 56.0 ± 8.8 days.; no significant difference was seen in the comparisons.

**Conclusions:**

The M_RBC_ can be estimated using iA1c or iA1c/GA in non‐diabetic individuals, and iA1c/GA in T2DM patients.

## Graphical abstract

We developed previously two methods for estimating mean erythrocyte age (M_RBC_). One method uses HbA1c and average glucose^20^ and the other uses erythrocyte creatine^14^. In this study, we propose two simple methods for estimating M_RBC_, using HbA1c and HbA1c/glycated albumin (GA).

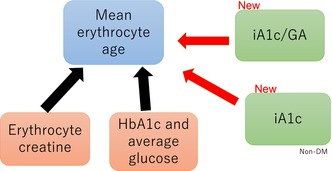

## 1 INTRODUCTION

The glycation of various proteins is higher in diabetic patients than in non‐diabetic individuals, suggesting that some glycated proteins may be responsible for the development and progression of diabetic complications.^1^ Among these glycated proteins, hemoglobin A1c (HbA1c) is widely used as an indicator of glycemic control for the treatment and diagnosis of diabetes in clinical settings.^2^ However, HbA1c is affected by erythrocyte lifespan, and its values are lower in patients with hemolytic anemia.^3^, ^4^ In contrast, glycated albumin (GA), another indicator of glycemic control, is not affected by erythrocyte lifespan, enabling accurate assessment of glycemic control status in patients with hemolytic anemia.^5^, ^6^, ^7^ In addition, we previously reported that the GA/HbA1c ratio is an indicator of hemolysis that is unaffected by glycemic control.^6^

Under normal conditions, the creatine content of human erythrocytes decreases with erythrocyte aging. Therefore, the erythrocyte creatine (EC) level has been shown to be high in patients with hemolytic anemia. The amount of creatine in packed erythrocytes has been reported to be an indicator of the mean erythrocyte age (M_RBC_).^8^, ^9^, ^10^ In addition, EC levels are elevated in patients with compensatory hemolysis, enabling the detection of hemolysis with higher sensitivity than reticulocytes or haptoglobin, which are conventional hemolysis indicators.^11^, ^12^ We have previously reported the usefulness of EC and M_RBC_ in diagnosing latent hemolysis.^13^

Conventionally, the erythrocyte lifespan is measured by labeling erythrocytes with a radioactive substance and assessing the decay curve of the labeled erythrocytes in the body. However, this technique is laborious and is seldom used. In Japan, the import of ^51^Cr, a radioactive substance, ceased in 2015, making it impossible to measure erythrocyte lifespan using this method. We have recently reported an equation to obtain M_RBC_ from EC based on the EC and erythrocyte lifespan determined using ^51^Cr reported by Fehr et al.^14^ This made it possible to determine the M_RBC_ using a single blood sample. However, the sale of reagents for measuring EC with high sensitivity has been discontinued, and high‐sensitivity EC measurements can only be performed in a limited number of laboratories that store these dyes. Therefore, we pursued a hemolytic indicator other than EC that reflect M_RBC_. We have also reported that EC and HbA1c had a high‐positive correlation in non‐diabetic individuals,^15^ and that EC and the HbA1c/GA ratio had a high‐positive correlation in individuals including patients with type 2 diabetes mellitus (T2DM).^16^

Measurement of erythrocyte lifespan is very important for hematological diseases, especially anemia. It would be useful to estimate the severity of hemolytic anemia, to determine the effects of treatment, and to detect latent hemolysis. This would also be useful for the differential diagnosis of anemia.

Based on these observations, in the present study, we formulated an equation to estimate the M_RBC_ in non‐diabetic individuals using HbA1c or the HbA1c/GA ratio. We also formulated an equation to estimate M_RBC_ in T2DM patients using the HbA1c/GA ratio.

## 2 METHODS

### 2.1 Patients

The present study included 238 participants, consisting of 107 non‐diabetic individuals, including 31 patients with hemolytic anemia, and 131 T2DM patients, including 11 with hemolytic anemia (Table 1).^16^ Hemolytic anemia included spherocytosis, autoimmune hemolytic anemia, and paroxysmal nocturnal hemoglobinuria. In addition, some patients with hemolysis who were older than 20 years and had laboratory data on complete blood counts and reticulocytes for clinical reasons were recruited.^15^ Patients with type 1 diabetes mellitus (T1DM), variant hemoglobin, or abnormal albumin metabolism, e.g., liver cirrhosis or overt proteinuria, or unstable glycemic control were excluded from the study. Hemolysis was defined as a clinical diagnosis of hemolysis or a haptoglobin level lower than the reference value. The study was approved by the institutional research board of each institute, and all participants provided written informed consent.

**TABLE 1:** Clinical characteristics and laboratory test results of non‐diabetic individuals (non‐DM) and patients with type 2 diabetes (T2DM).

Variable	Total	Non‐DM	T2DM	*p* *
*n*	238	107	131	–
Age (years)	60.9 ± 11.2	57.5 ± 12.9	63.8 ± 8.7	<0.0001
Male (%)	138 (58.0)	63 (58.9)	75 (57.3)	0.800
Hemolytic anemia (%)	42 (17.0)	31 (29.0)	11 (8.4)	<0.0001
Hb (g/dL)	13.3 ± 2.1	13.0 ± 2.5	13.5 ± 1.6	0.040
Reticulocytes (%)	0.50 ± 0.42	0.80 ± 0.37	0.20 ± 0.19	<0.0001
Haptoglobin (mg/dL)	32.2 ± 7.3	4.7 ± 7.5	67.7 ± 66.4	<0.0001
EC (μmol/g Hb)	2.04 ± 1.62	2.58 ± 2.19	1.61 ± 0.67	<0.0001
HbA1c (%)	6.5 ± 1.6	5.3 ± 0.9	7.5 ± 1.3	<0.0001
iA1c (mmol/mol)	47.5 ± 17.0	34.2 ± 10.0	58.3 ± 13.7	<0.0001
GA (%)	17.8 ± 5.5	13.4 ± 1.3	21.3 ± 5.1	<0.0001
iA1c/GA	2.68 ± 0.66	2.55 ± 0.74	2.79 ± 0.56	0.006

Abbreviations; non‐DM: non‐diabetic, T2DM: type 2 diabetes mellitus, Hb: hemoglobin, EC: erythrocyte creatine, A1C: HbA1c expressed in National Glycohemoglobin Standardization Program (NGSP) units, iA1c: HbA1c expressed in International Federation of Clinical Chemistry and Laboratory Medicine (IFCC) units, GA; glycated albumin.

* Non‐DM versus T2DM.

### 2.2 Laboratory methods

HbA1c levels were measured using high performance liquid chromatography in the National Glycohemoglobin Standardization Program (NGSP) units (A1C). The A1C values were converted into International Federation of Clinical Chemistry and Laboratory Medicine (IFCC) units (iA1c) using the formula developed by Hoelzel et al.^17^ GA levels were determined enzymatically using an albumin‐specific protease, ketoamine oxidase, and an albumin assay reagent (Lucica GA‐L; Asahi Kasei Pharma).^18^ Hematological examinations were carried out using a Sysmex SE 9000 (Sysmex), and reticulocyte counts were determined using a Sysmex R‐3000 (Sysmex). Haptoglobin was measured by an immunoturbidimetric assay using a Cobas c 501 (Roche Diagnostics). EC were assayed enzymatically as previously described.^11^, ^19^ Briefly, the plasma and buffy coat were aspirated from blood samples after centrifugation. After lysis with 0.1% saponin and deproteinization of packed erythrocytes with 0.15 M Ba(OH)_2_ and 0.15 M ZnSO_4_, the supernatant was obtained by centrifugation and filtration. Creatine concentration in the supernatant was measured using an enzymatic assay involving creatine amidohydrolase, sarcosine oxidase, and peroxidase. The measured data are expressed as micromoles per gram of hemoglobin (μmol/g Hb). Based on the EC values obtained from the participants, we estimated the EC‐M_RBC_ using Equation (1), as previously reported^14^:

(1) $$\mathrm{EC}-M_{\mathrm{RBC}}\;\left(\text{days}\right)=65.83–22.84\times \log_{e}\;\mathrm{EC}\;\left(\text{μmol}/g\;\mathrm{Hb}\right)$$

### 2.3 Statistical analysis

All data are shown as the mean ± standard deviation. For statistical analyses, the unpaired Student's *t*‐test, paired Student's *t*‐test, and χ^2^ test were used for comparisons between two groups, as appropriate. To analyze the correlation between two parameters, Pearson's correlation coefficient was calculated using the StatView computer program (Version 5.0, Abacus Concepts). *p* values were considered statistically significant at *p* < 0.05.

## 3 RESULTS

**FIGURE 1:**
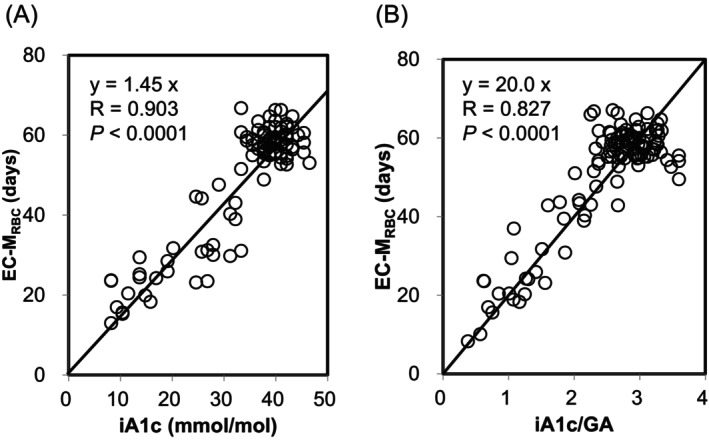
Correlations between the EC‐M_RBC_ and iA1c, and EC‐M_RBC_ and iA1c/GA in non‐diabetic individuals. Correlations between EC‐M_RBC_ and iA1c (A), and EC‐M_RBC_ and iA1c/GA (B) in 107 non‐diabetic individuals, including 31 patients with hemolytic anemia, are shown. The regression lines passing through the origin are indicated by solid lines.

In non‐diabetic individuals, the EC‐M_RBC_ showed a positive correlation with both iA1c and iA1c/GA levels with high‐correlation coefficients (EC‐M_RBC_ vs. iA1c: *R* = 0.903, *p* < 0.0001; EC‐M_RBC_ vs. iA1c/GA: *R* = 0.875, *p* < 0.0001; Figure 1). As the regression equations between the EC‐M_RBC_ and iA1c, and EC‐M_RBC_ and iA1c/GA passing through the origin were given as EC‐M_RBC_ = 1.45 × iA1c, and EC‐M_RBC_ = 20.0 × iA1c/GA, respectively, iA1c and iA1c/GA were used to estimate the iA1c‐M_RBC_ and iA1c/GA‐M_RBC_ in non‐diabetic individuals in the following equations:

(2) $$\mathrm{iA}1c-M_{\mathrm{RBC}}\;\left(\text{days}\right)=1.45\times \mathrm{iA}1c\;\left(\text{mmol}/\mathrm{mol}\right)$$

(3) $$\mathrm{iA}1c/\mathrm{GA}-M_{\mathrm{RBC}}\;\left(\text{days}\right)=20.0\times \mathrm{iA}1c/\mathrm{GA}\;\left(\text{mmol}/\mathrm{mol}/\%\right)$$

**FIGURE 2:**
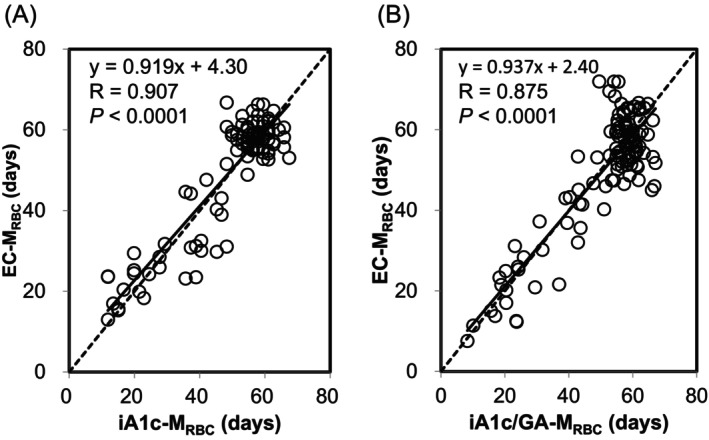
Correlations between the EC‐M_RBC_ and iA1c‐M_RBC_, and EC‐M_RBC_ and iA1c/GA‐M_RBC_ in non‐diabetic individuals. The iA1c‐M_RBC_ and iA1c/GA‐M_RBC_ were estimated using iA1c and iA1c/GA in 107 non‐diabetic individuals, including 31 patients with hemolytic anemia. Correlations between EC‐M_RBC_ and iA1c‐M_RBC_ (A), and EC‐M_RBC_ and iA1c/GA‐M_RBC_ (B) are shown. The regression lines for both parameters are shown as solid lines, and *y* = *x* is shown as a dashed line.

In non‐diabetic individuals, both iA1c‐M_RBC_ and iA1c/GA‐M_RBC_ showed strong positive correlations with EC‐M_RBC_ (EC‐M_RBC_ vs. iA1c‐M_RBC_: *R* = 0.907, *p* < 0.0001; EC‐M_RBC_ vs. iA1c/GA‐M_RBC_: *R* = 0.875, *p* < 0.0001; Figure 2). In addition, the regression lines between iA1c‐M_RBC_ and EC‐M_RBC_, and iA1c/GA‐M_RBC_ and EC‐M_RBC_ were nearly consistent with *y* = *x*.

Next, the estimation of the M_RBC_ in T2DM patients was examined. As HbA1c in T2DM patients is affected by glycemic control, M_RBC_ cannot be estimated from HbA1c. The EC‐M_RBC_ in non‐diabetic individuals did not differ significantly from that in T2DM patients (58.5 ± 3.4 days vs. 57.4 ± 5.2 days, *p* = 0.105), and there was no significant difference in iA1c/GA (2.94 ± 2.8 vs. 2.89 ± 0.45, *p* = 0.358). Therefore, the M_RBC_ in T2DM patients was estimated using the equation for the iA1c/GA‐M_RBC_ formulated for non‐diabetic individuals.

**FIGURE 3:**
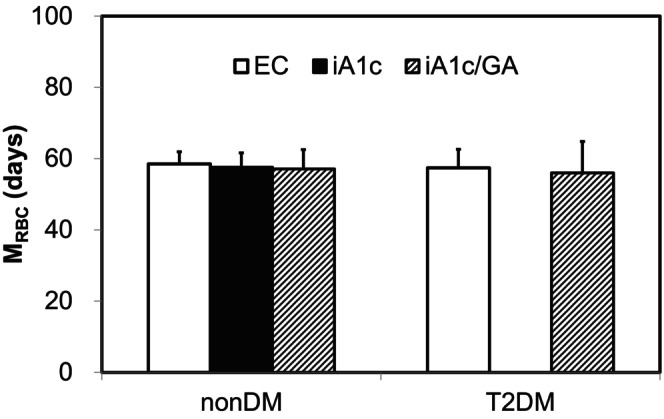
Comparisons of M_RBC_ values estimated from various indicators in non‐diabetic individuals without hemolytic anemia (non‐DM) and type 2 diabetic patients (T2DM) without hemolytic anemia. The results of the comparison of M_RBC_ values estimated from various indicators (EC, iA1c, and iA1c/GA) in 76 nondiabetic individuals (left) and120 T2DM patients (right) are shown. As HbA1c in T2DM patients is affected by glycemic control, M_RBC_ cannot be accurately estimated from HbA1c.

The EC‐M_RBC_, iA1c‐M_RBC_, and iA1c/GA‐M_RBC_ in non‐diabetic individuals without hemolytic anemia were 58.5 ± 3.4, 57.6 ± 4.0, and 57.1 ± 6.4 days, respectively, with no significant difference among the three parameters (Figure 3). In addition, the EC‐M_RBC_ and iA1c/GA‐M_RBC_ in T2DM patients were 57.4 ± 5.2 and 56.0 ± 8.8 days, respectively, with no significant difference between both parameters. Similar to EC‐M_RBC_, there was no significant difference in iA1c/GA‐M_RBC_ between non‐diabetic individuals and T2DM patients.

## 4 DISCUSSION

We demonstrated that the EC‐M_RBC_ had a strong positive correlation with iA1c and iA1c/GA in non‐diabetic individuals, and with iA1c/GA in T2DM patients. This method is useful for evaluating the severity of hemolytic anemia. Based on these findings, in the present study, we formulated equations to estimate M_RBC_ in nondiabetic individuals using iA1c and iA1c/GA, and in T2DM patients using iA1c/GA. A conventional method for estimating M_RBC_ involves the use of ^51^Cr‐labeled erythrocytes; however, this method is seldom used nowadays as it requires frequent blood collection, which is laborious and time‐consuming. Our method for estimating the M_RBC_ using HbA1c or the HbA1c/GA ratio requires only a single blood collection and is thus an easier and less costly method of estimating the M_RBC_.

As the M_RBC_ decreased, EC increased and HbA1c decreased in a mirror‐image fashion. EC was previously found to be highly correlated with M_RBC_, and was thus expected to reflect the M_RBC_.^10^ HbA1c showed a higher correlation with EC than other indicators of hemolysis, such as reticulocytes and haptoglobin; thus, HbA1c was also considered to be a potential indicator of M_RBC_. Based on these findings, we previously reported that HbA1c adjusted by EC accurately reflected the glycemic control in patients with hemolytic anemia.^16^

Based on our previously reported formula for estimating M_RBC_ from EC,^14^ we formulated an equation to estimate M_RBC_ using the regression formula of M_RBC_ and HbA1c obtained from EC in non‐diabetic individuals with simultaneous measurements of both EC and HbA1c. HbA1c expressed in NGSP units (A1C) contains approximately 2.15% other substances.^17^ By contrast, HbA1c expressed in IFCC units (iA1c) is a pure measure of HbA1c. Thus, the regression line between EC‐M_RBC_ and A1C did not pass through the origin, whereas that between EC‐M_RBC_ and iA1c‐M_RBC_ passed through the origin. Therefore, iA1c‐M_RBC_ can be estimated by multiplying iA1c with a constant.

In this study, we proposed the equation iA1c‐M_RBC_ = 1.45 × iA1c [Equation (2)], and validated the constant. We have previously described the following equation for estimating the M_RBC_ based on HbA1c and the mean blood glucose (MBG) level obtained from continuous glucose monitoring^20^:

(4) $$M_{\mathrm{RBC}}\;\left(\text{days}\right)=\mathrm{iA}1c\;\left(\text{mmol}/\mathrm{mol}\right)/\left[\left(1000–2/3\times \mathrm{iA}1c\right)\times k_{g}\times \mathrm{MBG}\;\left(\mathrm{mg}/\mathrm{dL}\right)\right]$$

The “2/3 × iA1c” term in the above equation is much smaller than 1000, so the omission of this term does not greatly affect the values. In addition, an MBG level of 100 mg/dL can be used in non‐diabetic individuals. A subsequent analysis determined that *k*_g_ in Equation (4) was 7.0 × 10^−6^.^21^ Therefore, the following equation was derived from Equation (4):

(5) $$\;\begin{array}{cc} M_{\mathrm{RBC}}\;\left(\text{days}\right) & =\mathrm{iA}1c\;\left(\text{mmol}/\mathrm{mol}\right)/\left(1000\times k_{g}\times 100\right)\\ & =\mathrm{iA}1c/\left(7.0\times 10^{-6}\times 10^{5}\right)\\ & =\mathrm{iA}1c/0.7=1.43\times \mathrm{iA}1c \end{array}$$

This derived equation is almost the same as our proposed equation, M_RBC_ = 1.45 × iA1c. Because HbA1c is affected by glycemic control in diabetic patients, M_RBC_ cannot be estimated from HbA1c in diabetic patients. Therefore, we attempted to estimate the M_RBC_ from the HbA1c/GA ratio. Although HbA1c is affected by glycemic control, by adjusting for the effect of GA on HbA1c, the HbA1c/GA ratio can be an indicator of hemolysis unaffected by glycemic control.^6^ Given that there was no significant difference in iA1c/GA between non‐diabetic individuals and T2DM patients, the iA1c/GA‐M_RBC_ was estimated in T2DM patients using the equation used to derive the equation for estimating M_RBC_ from iA1c/GA, based on the correlation between EC‐M_RBC_ and iA1c/GA in non‐diabetic individuals. The results showed values similar to those of EC‐M_RBC_. Therefore, M_RBC_ in T2DM patients could be estimated using the iA1c/GA.

Can our proposed equation for estimating M_RBC_ using HbA1c be applied to all individuals without diabetes? The answer is no. In cases of hemoglobin variants, it is difficult to accurately measure HbA1c; therefore, M_RBC_ cannot be estimated in such cases. Abnormal GA levels, which may result from clinical conditions, such as liver cirrhosis, overt proteinuria, thyroid dysfunction, and glucocorticoid administration,^5^ hinder the accurate estimation of M_RBC_ using iA1c/GA. As GA is affected by plasma glucose fluctuations, the GA/HbA1c ratio in T1DM patientswith large plasma glucose fluctuations is higher than that in T2DM patients.^22^ In the present study, as the equation for estimating M_RBC_ was formulated using iA1c/GA in T2DM patients, this equation cannot be applied to T1DM patients. Further studies are needed to evaluate M_RBC_ in T1DM patients. In addition, GA is known to reflect more recent glycemic control than HbA1c.^5^ Therefore, the iA1c‐M_RBC_ and iA1c/GA‐M_RBC_ cannot be estimated in patients with rapid deterioration or improvement in glycemic control.

In conclusion, M_RBC_ can be easily estimated using iA1c or iA1c/GA in non‐diabetic individuals, and iA1c/GA in T2DM patients. The results of this study are expected to facilitate further studies on M_RBC_ in various hematological diseases.

## AUTHOR CONTRIBUTIONS

MKo researched the data, and literature, wrote the first draft of the manuscript, contributed to the discussions, and edited the manuscript. MKa researched the data, contributed to the discussions, and edited the manuscript. TO researched the data, reviewed the literature, conceived the study, contributed to the discussion, and edited the manuscript. All authors approved the final version of the manuscript.

## FUNDING INFORMATION

This study was partly supported by Asahi Kasei Pharma Corporation.

## CONFLICT OF INTEREST STATEMENT

T.O. received research funding from Asahi Kasei Pharma. M.Ko, and M.Ka declare no conflicts of interest.

## ETHICAL APPROVAL AND CONSENT TO PATIENTS

This study was approved by the Institutional Research Board of each institution. All samples were prepared and analyzed in accordance with the protocols approved by the institutional responsible committee at Kumamoto University and other collaborating institutions. Patients with type 2 diabetes mellitus and healthy volunteers were provided with detailed information about the study, and all participants provided written informed consent.

## Data Availability

The data that support the findings of this study are not publicly available.
